# TCGA Molecular Prognostic Groups of Endometrial Carcinoma: Current Knowledge and Future Perspectives

**DOI:** 10.3390/ijms231911684

**Published:** 2022-10-02

**Authors:** Damiano Arciuolo, Antonio Travaglino, Antonio Raffone, Diego Raimondo, Angela Santoro, Daniela Russo, Silvia Varricchio, Paolo Casadio, Frediano Inzani, Renato Seracchioli, Antonio Mollo, Massimo Mascolo, Gian Franco Zannoni

**Affiliations:** 1 Pathology Unit, Department of Woman and Child’s Health and Public Health Sciences, Fondazione Policlinico Universitario Agostino Gemelli IRCCS, 00168 Rome, Italy; 2 Pathology Institute, Catholic University of Sacred Heart, 00168 Rome, Italy; 3 Pathology Unit, Department of Advanced Biomedical Sciences, University of Naples “Federico II”, 80138 Naples, Italy; 4 Division of Gynaecology and Human Reproduction Physiopathology, Department of Medical and Surgical Sciences (DIMEC), IRCCS Azienda Ospedaliera Univeristaria di Bologna. S. Orsola Hospital, University of Bologna, 40126 Bologna, Italy; 5 Gynecology and Obstetrics Unit, Department of Medicine, Surgery and Dentistry “Schola Medica Salernitana”, University of Salerno, 84081 Baronissi, Italy

**Keywords:** TCGA, endometrial carcinoma, mismatch-repair, p53, molecular, prognosis, treatment, histotype, POLE, microsatellite

## Abstract

The four TCGA-based molecular prognostic groups of endometrial carcinoma (EC), i.e., POLE-mutant, mismatch repair (MMR)-deficient, p53-abnormal, and “no specific molecular profile” (NSMP), have recently been integrated into ESGO-ESTRO-ESP guidelines. The POLE-mutant and MMR-deficient groups are associated with high mutational load, morphological heterogeneity, and inflammatory infiltration. These groups are frequent in high-grade endometrioid, undifferentiated/dedifferentiated, and mixed histotypes. POLE-mutant ECs show good prognosis and do not require adjuvant treatment, although the management of cases at stage >II is still undefined. MMR-deficient ECs show intermediate prognosis and are currently substratified based on clinicopathological variables, some of which might not have prognostic value. These groups may benefit from immunotherapy. P53-mutant ECs are typically high-grade and often morphologically ambiguous, accounting for virtually all serous ECs, most carcinosarcomas and mixed ECs, and half of clear-cell ECs. They show poor prognosis and are treated with chemoradiotherapy; a subset may benefit from HER2 inhibitors or PARP inhibitors. The NSMP group is the most frequent TCGA group; its prognosis is highly variable and affected by clinicopathological/molecular factors, most of which are still under evaluation. In conclusion, the TCGA classification has improved diagnosis, risk stratification, and management of EC. Further studies are needed to resolve the points of uncertainty that still exist.

## 1. Introduction

The Cancer Genome Atlas (TCGA) Research Network has revolutionized our approach to endometrial carcinoma (EC). For decades, the risk stratification of EC has been based on histopathological features, such as tumor grade and histotype, depth of myometrial invasion, and cervical and adnexal involvement. In 2013, an integrated molecular characterization of EC by TCGA showed that EC could be subdivided into four prognostically relevant groups based on mutational burden and somatic copy-number variations [1,2,3,4]. Subsequent studies have found that cheaper immunohistochemical and molecular tests can serve as surrogates of the complex and expensive analyses by TCGA [4,5,6,7,8]. The four molecular prognostic groups identified by surrogate tests are POLE-mutated, mismatch repair (MMR)-deficient, p53-abnormal, and “no specific molecular profile” (NSMP) (Table 1). These groups have now been integrated into the European (ESGO-ESTRO-ESP) guidelines for management of EC [9]. However, several issues must still be resolved, such as the significance of the four groups across different histotypes and tumor stages, as well as the value of novel immunohistochemical and molecular prognostic markers [4].

In this review, we deal with each molecular prognostic group, discussing their clinicopathological and molecular features, their significance across different histotypes, their possible integration with additional prognostic markers, and their possible predictive value for novel treatments.

## 2. POLE-Mutated

The POLE-mutated group was the least common molecular group in the TCGA series (7.3% of all ECs) and was labeled “ultramutated group” based on its exceptionally high mutational burden, (232 × 10^−6^ mutations per megabase). The authors noted that all and only ultramutated ECs showed pathogenetic mutations in the exonuclease domain of Polymerase-ε (POLE), which thus served as a surrogate of the ultramutated status [1] (Table 1). POLE-mutant tumors are characterized by an overwhelmingly favorable prognosis (progression-free survival of 92% to 100% [1,5,6,7,8,10]). Compared with other TCGA groups, POLE-mutant ECs show younger age (mean 58.6 years), lower BMI (mean 27.2), and an earlier FIGO stage (stage I in 93.7% of cases) [11] (Table 2).

More than 80% of pathogenetic POLE mutations fall into one of five hotspots (P286R, V411L, S297F, A456P, S459F); furthermore, mutations in other sites within the exonuclease domain are rarer and are pathogenetic in about 39% of cases. Mutations outside the exonuclease domain are pathogenetic in only 4% of cases [10]. Unlike the MMR-deficient and p53-abnormal groups, there are no immunohistochemical surrogate markers to identify the POLE-mutated group [4,5,6,7,8].

The TCGA series only included endometrioid and serous ECs, and all POLE-mutant ECs were of the endometroid type. Interestingly, in apparent contrast with their good prognosis, about half of POLE-mutant ECs were high-grade [1]. POLE mutations are indeed significantly more frequent in high-grade endometrioid carcinoma (12.1%) than in low-grade endometrioid carcinoma (6.2%) [12]. Morphological heterogeneity and marked atypia are common features in POLE-mutant ECs, which may also show giant anaplastic cells [13,14,15]. This has suggested that most POLE-mutant ECs are currently overtreated based on their histological appearance [16].

Subsequent studies have shown that POLE-mutations may be found in several other EC histotypes [13,17,18,19,20,21,22,23,24,25,26,27,28,29,30]. A relatively high frequency of POLE-mutations was found in undifferentiated/dedifferentiated carcinoma (12.4%) [31], while a low frequency was found in clear-cell carcinoma (3.8%) [32] and carcinosarcoma (5.3%) [33] (Table 3).

Remarkably, POLE mutation was found in a significant proportion of mixed endometrioid-serous carcinomas arising in young women (16%); furthermore, these tumors are thought to arise as endometrioid ECs that secondarily develop a serous morphology, with or without p53 mutations [34]. Gynecological pathologists agree that POLE-mutant ECs with a serous morphology and immunophenotype should be deemed as endometrioid carcinomas [4,15,34,35]. In these tumors, both the high-grade features and the p53 mutation are consequence of the high mutational load and have no clinical significance. Similarly, the presence of MMR deficiency in POLE-mutant ECs seems to have no prognostic value. Therefore, POLE mutations prognostically supersede both MMR deficiency and p53 mutations [4,10,36]. Even histotype seems to have no value in the case of POLE mutation, since POLE-mutant non-endometrioid ECs still show a good prognosis [9,18,37,38,39]. The mutational load itself causes a strong immune response due to the exposition of many neoantigens, which is reflected in a lymphocytic infiltration that accompanies most (~79%) POLE-mutant ECs and could be responsible for their good prognosis [13,40]. However, Talhouk et al. found that the immune response was not independently associated with prognosis, suggesting that other factors drive prognosis in POLE-mutant ECs [41].

The POLE-mutated group was found to be the least prognostically affected by clinicopathological factors [42]. This is the reason why the ESGO-ESTRO-ESP guidelines consider POLE-mutant EC up to FIGO stage II as low-risk tumors that do not need adjuvant treatment [9]. This approach has been criticized by some authors due to the lack of prospective studies supporting it. The authors also highlighted that >10% of POLE-mutant ECs present at a FIGO stage >II, and it is unclear how these cases should be treated [43]. Given the prominent lymphocytic infiltrate found in most POLE-mutant ECs, it is reasonable to hypothesize that these tumors may benefit from immunotherapy [41].

## 3. MMR-Deficient

The MMR-deficient group was first defined by TCGA as “hypermutated group”, since it showed a high mutational load (18 × 10^−^^6^ mutations per megabase), which was, however, lower than that of the ultramutated/POLE-mutated group. The hypermutated group was the second most common group after the NSMP group, accounting for 28% of EC cases [1]. All hypermutated ECs showed high microsatellite instability, a condition associated with the rapid accumulation of genomic mutations [1,44]. Since microsatellite instability is typically caused by a deficiency in the MMR system, immunohistochemistry for MMR proteins has been used as a surrogate test to identify the hypermutated group (Table 1). The four MMR proteins assessed are MLH1, MSH2, MSH6, and PMS2. These proteins form two heterodimers, which are MLH1-PMS2 and MSH2-MSH6. When MLH1 or MSH2 are lost, there is a consequent loss of PMS2 or MSH6, respectively. On the other hand, a loss of MSH6 or PMS2 expression can occur as an isolated event. For this reason, the immunohistochemical assessment of only MSH6 and PMS2 has been suggested to have the same accuracy as the full MMR panel in identifying MMRd cases [45]. The assessment of MMR immunohistochemical expression may be difficult and affected by fixation issues. A positive internal control in endometrial stroma and lymphocytes should be clearly evaluable. A normal, retained expression of MMR proteins consists of positivity in tumor cell nuclei, which should be stronger than stromal positivity [46].

Histologically, the MMR-deficient group showed several similarities with the POLE-mutated group. In fact, the MMR-deficient group are mostly endometrioid (85.8%), with a high frequency of high-grade cases (47.4%) [16] (Table 2). MMR deficiency is significantly more frequent in high-grade than in low-grade endometrioid carcinoma (39.7% vs. 24.7%) [21] and is particularly frequent in undifferentiated/dedifferentiated carcinoma (44%) [31] and in mixed ECs with an endometrioid component (16–66%) [30,34,47]. On the other hand, the frequency is lower in clear-cell carcinoma (9.8%) [32] and carcinosarcoma (7.3%) [33] (Table 3). As discussed for POLE-mutant ECs, MMR-deficient ECs with a serous morphology and/or immunophenotype are considered to be endometrioid EC [4,15,34,35]. MMRd ECs often show a prominent lymphocytic infiltration and striking morphological heterogeneity [15].

The overall prognosis of MMR-deficient ECs is intermediate. MMR deficiency prognostically supersedes p53 abnormalities but is superseded by POLE mutations [4,10,36]. Compared to POLE-mutant ECs, MMR-deficient ECs seem to be more affected by clinicopathological variables, although not as much as NSMP ECs [42]. The ESGO-ESTRO-ESP guidelines substratify MMR-deficient ECs into different risk groups based on FIGO grade, histotype, depth of myometrial invasion, and LVSI [9]. However, it is unclear whether these factors are all prognostically significant in MMR-deficient ECs [4]. For instance, while deep myometrial invasion and LVSI significantly worsen the prognosis of MMR-deficient ECs, tumor grade seems not to have independent prognostic value [48]. It has been suggested that even histotype has no prognostic value in MMR-deficient ECs [4]. In fact, MMR deficiency seems to be consistently associated with an intermediate prognosis across different histotypes, leading to worsened outcomes in early-stage, low-grade ECs [49], and improved outcomes in non-endometrioid ECs [8,38,50]. According to this view, differences in grade and histotype might be part of the morphological heterogeneity of MMR-deficient ECs, with no impact on prognosis [4]. Undifferentiated/dedifferentiated carcinoma is an exception. About 2/3 of undifferentiated/dedifferentiated carcinoma show the loss of one of three crucial proteins of the SWI/SNF complex, i.e., ARID1B, SMARCA4/BRG1, and SMARCB1/INI1. SWI/SNF-deficient carcinomas have shown an exceedingly bad prognosis, even in the presence of an MMR-deficient signature [37]. Interestingly, it has been suggested that MMR-deficient ECs associated with MLH1 promoter methylation have a poorer prognosis than MMR-deficient EC associated with mutations in the MMR genes [37]. However, it is unclear whether this difference might require a different treatment.

With regard to therapy, MMR-deficient ECs have shown higher susceptibility to radiotherapy than MMR-proficient ECs [51]. Based on their high mutational load and immune infiltrate, MMR-deficient ECs are a candidate for immunotherapy [52].

## 4. p53-Abnormal

After the exclusion of ultramutated and hypermutated tumors, TCGA subdivided ECs with a low mutational load into two groups based on somatic copy-number variation: “copy number—high” and “copy number—low”. The copy number—high group was characterized by a high frequency of TP53 mutation (85%) and serous morphology (73.3%) and was therefore defined as the “serous group” [1]. Such a group represents the prototypical “type II” EC as it is associated with older age, non-endometrioid morphology, an advanced stage, and poor prognosis [1,11,16,42] (Table 2).

Since p53 immunohistochemistry has been used as a cheaper surrogate of TP53 molecular testing, the copy number—high/serous group has subsequently been termed as the p53-abnormal group [4,5,6,7,8,9] (Table 1). The systematic assessment of p53 immunohistochemical expression in TP53-mutant tumors has shown three possible aberrant patterns of p53: overexpression (strong expression in >70–80% of tumor cell, accounting for 85.6% of cases); complete loss (11.5% of cases); and cytoplasmic expression (1.9% of cases) [53]. An optimized immunohistochemical protocol is crucial to correctly identify these patterns. For example, tumors with low proliferation may show a very focal and faint p53 positivity, which can be misinterpreted as a “complete loss” pattern, especially when a positive internal control is not clearly assessable [54,55]. The presence of a subclonal p53-abnormal pattern is often associated with MMR deficiency or POLE mutation [53]; in the presence of these signatures, p53 abnormalities have no prognostic value [36]. A small subset of TP53-mutant tumors (~5%) do not show abnormalities in p53 expression and thus cannot be identified by immunohistochemistry [54]. In addition, there is a subset of copy number—high ECs that do not show TP53 mutations and can only be classified by a molecular analysis of copy-number variations [1]. Despite not being a perfect surrogate of copy-number analysis, p53 immunohistochemistry has shown sufficient accuracy to be used in the common practice [4,5,6,7,8].

P53-abnormal tumors are typically high-grade and show striking nuclear atypia [15]. In fact, a p53-abnormal signature is by far more common in high-grade than in low-grade endometrioid ECs (21.3% vs. 4.7%) [12]. A recent study suggested that p53-abnormal low-grade endometrioid ECs can be observed in elderly patients [56]. A p53-abnormal signature is virtually present in all serous ECs [4]; furthermore, morphologically serous ECs with POLE-mutant or MMR-deficient signatures are considered as serous-like, high-grade endometrioid ECs, as discussed above. The p53-abnormal group accounts for the vast majority of carcinosarcomas (73.9%), which commonly arise from serous ECs [33], and almost half of clear-cell ECs (42.5%) [32] (Table 3). The p53-abnormal signature is often associated with ambiguous morphology and is frequent in mixed ECs with a serous component and in ambiguous ECs [15,34,35,57].

The biological behavior of p53-abnormal ECs is consistently aggressive across different histotypes [4,5,6,7,8,38,39,42]. ESGO-ESTRO-ESP guidelines include all p53-abnormal ECs in the high-risk group (except for non-myoinvasive cases) [9]. It has been suggested that prognostic differences do exist among p53-abnormal tumors. For instance, serous carcinoma may be more aggressive than p53-abnormal endometroid carcinoma but less aggressive than carcinosarcoma [39,58]. However, these differences have not consistently been reported and it is unclear if they are such as to need different treatments. Moreover, differentiating between serous carcinoma and p53-abnormal endometrioid carcinoma can be difficult, especially considering the morphological ambiguity that often accompanies p53-abnormal ECs. Therefore, it appears appropriate to lump all p53-abnormal ECs together in the same risk group [4,15,59].

Regarding treatment, p53-abnormal ECs always need adjuvant treatment. In all myoinvasive cases, chemoradiotherapy is indicated [9]. HER2 amplification has been identified as a therapeutic target in a subset of p53-abnormal carcinomas, regardless of the histotype [60,61,62,63]. Frequent high DNA damage and high PARP-1 expression have also been observed p53-abnormal ECs, suggesting the possibility to use PARP inhibitors [64].

## 5. NSMP

The remaining TCGA group showed neither high mutational load nor significant copy-number variations. Such group was deemed “copy number—low/endometrioid group” and was considered to represent the prototypical type I EC [1]. Since this group is identified by the absence of the molecular signatures of the other groups, it has been deemed “NSMP” [4,9] (Table 1). The NSMP group is the most frequent TCGA group (~40% of cases) at intermediate prognosis, and is similar to the MMRd group [1,4,5,6,7,8].

While the vast majority (84.4%) of NSMP ECs are low-grade endometrioid tumors [16] (Table 2), the NSMP group can be found in almost any EC histotype, accounting for all mesonephric-like ECs (100%) [65,66], almost half of clear-cell ECs (40.9%) [31], and sizable quantities of neuroendocrine ECs (36%) [30], high-grade endometrioid ECs (28%) [12], undifferentiated/dedifferentiated ECs (25%) [31], and carcinosarcomas (13.5%) [33]. The NSMP group is virtually never found in serous EC; however, serous ECs with a “copy number—high” signature may lack abnormal immunohistochemical expression of p53 and even TP53 mutations, resulting in a NSMP classification. These cases are considered biologically and prognostically analogous to p53-abnormal serous ECs [1,53,54]. Mixed ECs typically are not NSMP, although data in this regard are based on small series [67] (Table 3).

The ESGO-ESTRO-ESP guidelines recommend substratifying the NSMP group based on the same criteria as the MMR-deficient group [9]. However, there is evidence suggesting that the NSMP is more prognostically heterogeneous and more heavily affected by other clinicopathological factors than the MMR-deficient group [4]. In fact, NSMP non-endometrioid ECs showed a bad prognosis similar to that of p53-abnormal ECs [8,38,39,65,66], while NSMP endometrioid ECs showed a highly heterogeneous prognosis, ranging from as good as POLE-mutant ECs to as bad as p53-abnormal ECs [1,8,14,58,68]. Several authors proposed a possible substratification of the NSMP group based on histological, immunohistochemical, and molecular markers, some of which may constitute therapeutic targets [8,64,69,70]. The Leiden group found that NSMP endometrioid ECs could be subdivided into three groups: high-risk, intermediate-risk, and low-risk. High-risk cases showed LVSI and/or overexpression of L1CAM (positivity in ≥10% of tumor cells). Intermediate-risk cases lacked LVSI and L1CAM overexpression but showed CTNNB1 exon 3 mutations. Low-risk cases were CTNNB1 wild-type with no LVSI or L1CAM overexpression [8]. Such subclassification of NSMP cases is currently under evaluation in the PORTEC-4a study [69]. Other proposed substratifications are based on the presence of DNA damage biomarkers [64] and the status of several genes such as PTEN, AKT, PI3KCA, PI3KR1, and KRAS [70]. All these findings highlight how the NSMP group is a heterogenous admixture of clinically and molecularly different entities, which require different management.

## 6. Conclusions

The TCGA classification has offered the possibility to improve the risk stratification and management of EC. Grouping ECs based on molecular signatures may help reduce inter- and intraobserver variability in the assignment of grades and histotypes, especially for morphologically heterogeneous and ambiguous ECs. Moreover, specific therapeutic possibilities can be found in each molecular group. Further research is needed to resolve the issues that still exist, such as the substratification of the NSMP group, the prognostic value of clinicopathological variables in MMR-deficient ECs, and how to treat advanced POLE-mutant ECs.

## Figures and Tables

**Table 1:** Definition of the 4 molecular prognostic groups of endometrial carcinoma.

Molecular Prognostic Group	Original Name	Identified by	Surrogate Marker
POLE-mutated	POLE/ultramutated	High mutational load (232 × 10^−6^ mutations per megabase)	POLE exonuclease domain mutation
MMR-deficient	MSI/hypermutated	High mutational load (18 × 10^−6^ mutations per megabase)	Loss of MMR proteins expression
p53-abnormal	Copy number—high/serous	Low mutational load; high copy-number variations	Abnormal p53 expression
NSMP	Copy number—low/ endometrioid	Low mutational load; low copy-number variations	Absence of the other markers

**Table 2:** Clinico-pathological features of the 4 molecular prognostic groups of endometrial carcinoma.

Molecular Prognostic Group	Age	BMI	Stage >I	High Grade	Non- Endometrioid Histotype	LVSI	Deep Myometrial Invasion	Lymph Node Involvement
POLE-mutated	58.5 ± 2.7	27.2 ± 0.9	6.3%	39.6%	13.9%	32.7%	27.3%	0%
MMR-deficient	66.5 ± 0.6	30.6 ± 1.2	27.4%	47.4%	14.2%	41.3%	44.5%	9.9%
p53-abnormal	71.1 ± 0.5	29.1 ± 0.5	49.2%	90%	73%	13.8%	48.9%	23.7%
NSMP	64.2 ± 1.9	32.3 ± 1.4	19.5%	15.6%	3.3%	48.8%	27.4%	4.3%

**Table 3:** Prevalence of the 4 TCGA molecular prognostic groups across different histotypes of endometrial carcinoma.

Molecular Prognostic Group	LG-EEC	HG-EEC	SC	CCC	Mixed	UDC/ DDC	CS	NEC ***	MLC
POLE-mutated	6.2%	12.1%	0% *	3.8%	5.6%	12.4%	5.3%	7.1%	0%
MMR-deficient	24.7%	39.7%	0% *	9.8%	33.3%	44%	7.3%	42.9%	0%
p53-abnormal	4.7%	21.3%	100% **	42.5%	61.1%	18.6%	73.9%	35.7%	0%
NSMP	63.5%	28%	0% *	40.9%	0%	25%	13.5%	14.3%	100%

LG-EEC: low-grade endometrioid carcinoma; HG-EEC: high-grade endometrioid carcinoma; SC: serous carcinoma; CCC: clear-cell carcinoma; Mixed: mixed carcinoma; UDC/DDC: undifferentiated/dedifferentiated carcinoma; CS: carcinosarcoma; NEC: neuroendocrine carcinoma; MLC: mesonephric-like carcinoma. * Endometrial carcinomas with a serous morphology and POLE mutation or MMR deficiency are diagnosed as serous-like high-grade endometrioid carcinoma. ** Serous carcinomas with normal p53 expression in the presence of TP53 mutation, or with no TP53 mutation but with high copy-number variation, may rarely occur. *** The only published series of endometrial neuroendocrine carcinoma assessed with the TCGA classifier was constituted of 4 pure neuroendocrine carcinomas and 10 mixed carcinomas with a neuroendocrine component [30].

## Data Availability

Not applicable.
